# A Protest against Indiscriminate Meatus-Cutting

**Published:** 1887-03

**Authors:** J. W. S. Gouley

**Affiliations:** Surgeon to Bellevue Hospital, New York


### A PROTEST AGAINST INDISCRIMINATE MEATUS-CUTTING.

BY J. W. S. GOULEY, M. D.,

Surgeon to Bellevue Hospital, New York.

Urethral strictures of the balanic region, whether congenital or due to injury, or to disease, are well known to be of very frequent occurrence, and to be often accompanied with dysury, vesical irritation, "reflex neuroses," and other symptoms; but, of late years, the import of these consequences has been greatly over-estimated, and this has too often led to rash and unwarranted surgical interferences.

Meatus-cutting, or, to give the operation a proper technical name, *porotomy*, has become the fashion, and every adolescent and adult, who is not inflicted with congenital hypospadias, must have his meatus cut, for he is told that the nozzle of his urine-hose should be of greater calibre than the hose itself. He submits to this medical decree, however contrary he knows it to be to general physical and mechanical laws; and inasmuch as nearly every tyro in medicine also accepts this view, he is ready to undertake the mutilation—for what surgical operation is of easier performance than porotomy? The consequence is that there are at this moment, throughout the vast country, thousands of men who cannot urinate while standing without wetting their shoes, and who are suffering from other inconveniences due to traumatic bolanic hypospadias, and there are already surgeons undertaking operations for the repair of the deformity, as was the case among gynæcologists after the uterus-cutting craze had died out.

The doctrine that the meatus should be the largest part of the urethra is not only unsound but most dangerous, and is leading to much evil to sufferers and physicians. It is, therefore, high time to protest against the indiscriminate performance of porotomy, and particularly against those incisions which result in deformity of the urethra.

The congenitally narrow urinary meatus is very often encountered, yet comparatively few patients are ever inconvenienced by this defect. Many elderly men who seek the surgeon's advice on account of prostatic obstruction to urination are surprised when told that the external urethral orifice is abnormally narrow, and say that they never suffered in consequence of this abnormity, which they had not before regarded as such, and that until came the prostatic obstruction, the bladder was always easily and quickly emptied. In a considerable number of such cases the meatus barely admits a catheter of the diameter of three and four millimetres. Of course, here it is not necessary to enlarge the meatus by incision to a degree sufficient to permit the easy passage of suitable catheters or of a fair-sized lithotrite, should the presence of a vesical stone render necessary the use of such an instrument, but to incise through and through the whole balanic region is, to say the least, as unwarranted as unsurgical.

It not unfrequently happens that surgeons are consulted by middle-aged men, with congenital stricture at the meatus, who, having contracted their first urethritis, are for the first time inconvenienced by the narrowness of the urethral orifice. In these cases, the urethritis lingers in the balanic region, and does not resolve, as does generally the first attack, but becomes chronic. Four months thereafter continues the purulent discharge, the glans penis is indurated, the lips of the meatus are red and swollen, and urination is more or less impeded. The surgeon's skill being at last invoked, he forthwith decides to perform the operation of porotomy, which he follows by catheterism of the balanic region once every second day, until cicatrization is complete, and thus obtains a radical cure of the chronic urethritis and of the congenital stricture. But for this attack of urethritis the patient might never have known that he had an abnormally narrow meatus. Many practicers of medicine can tell a similar story.

Strictures of the balanic region are not ordinarily amenable to treatment by dilatation, but require incision, which is the most prompt and efficient method that can be employed for their eradication; but the incision should be directed and proportioned in accordance with the size and conformation of the glans penis and the condition of the extremity of the urethra. When, for instance, the meatus is normally situated, a sufficiently free, central longitudinal cut along the floor of the urethra answers the purpose of simply enlarging, within proper limits, the contracted urethral extremity; butwhen there happens to be a slight balanic congenital hypospadias, this kind of incision only increases the deformity and fails to relieve the stricture, which can be successfully treated only by bilateral porotomy, performed in such a way as not to increase the hypospadias.

The object of these extremely free incisions of the urinary meatus seems to be to enable those who practice them to introduce instruments of very large calibre through strictures, real or imaginary, of the deeper parts of the urethra. This indiscriminate over-dilatation of the urethra is another of the many prevalent surgical heresies. What is the ostensible reason for thus overstretching the urethra? It is that the stricture or strictures may not recur. Let those enthusiasts follow their cases, and they will soon find that there are strictures that recur after any and all methods of treatment, while the majority are curable by simple dilatation, and sometimes in spite of their mutilations, and a few of these latter, and only these few, can they reckon among their cures. Now, it is asked, what are the results of frequently introducing sounds of the diameters of eleven, twelve, and thirteen millimetres into the average human urethra? The most careful observation of many cases so treated shows that while the stricture, in some instances, does not recur, the urethra as an organic channel is entirly spoiled; it becomes to a normal urethra what an old, worn-out, hardened, and entirely inelastic India-rubber tube is to one which has just come from the workman's hands. The urethra which is often over-distended by sounds or catheters soon loses a very considerable number of its mucous follicles, becomes dry, leathery, inelastic, patulous, and no longer capable of successfully propelling the urine, which at length slobbers out of the wide urethral orifice, instead of being forced, as it should be, in a well-formed stream, through a narrow outlet.

It is said that the genital functions also are impaired by this over-distention of the urethral canal.

Is it, therefore, ever justifiable to over-distend the whole urethra, and to maintain the canal in that state of over-distention? Most assuredly it is not rational or proper to do so. Useful instruments have been devised to obviate the evil of over-stretching the whole canal. These instruments are so constructed as to over-distend, when deemed necessary, the strictured part only of the urethra, and save injury to the normal part of the canal, but they are little used. Dilating instruments of this sort should be occasionally employed during the treatment of strictures of the deep urethra, but the main object of moderate dilating catheterism is to restore the urethra, as nearly as possible, to its normal suppleness. Overstretching is here a traumatism which, when frequently repeated, is certain to destroy the suppleness and elasticity of the urethra, and render it almost useless as an organ of the human body.—*Med. Register.*

